# Genetic variants in myostatin and its receptors promote elite athlete status

**DOI:** 10.1186/s12864-023-09869-2

**Published:** 2023-12-11

**Authors:** Agata Leońska-Duniec, Małgorzata Borczyk, Michał Korostyński, Myosotis Massidda, Ewelina Maculewicz, Paweł Cięszczyk

**Affiliations:** 1 Faculty of Physical Education, Gdansk University of Physical Education and Sport, Gdansk, 80-336 Poland; 2 Department of Medical Sciences and Public Health, University of Cagliari, Cagliari, 09124 Italy; 3 Laboratory of Pharmacogenomics, Department of Molecular Neuropharmacology, Maj Institute of Pharmacology, Polish Academy of Sciences, Cracow, 31-343 Poland; 4 Faculty of Physical Education, Jozef Pilsudski University of Physical Education in Warsaw, Warsaw, 00-809 Poland

**Keywords:** Genome sequencing, Candidate gene, Sports genetics, Myostatin, Sports skills

## Abstract

**Background:**

While product of the myostatin gene (*MSTN*) is an important factor influencing muscle growth, which is well confirmed in nonhuman species, it has not been clearly confirmed whether *MSTN* expression influences interindividual differences in skeletal muscle mass, affects posttraining changes, or plays a role in the age-related loss of muscle mass and function in humans. Although the inconclusive results are usually explained by ethnic differences and the low frequency of some alleles, it is possible that the role of receptors (ACVR2A and ACVR2B) that affect the biological activity of myostatin is crucial. Therefore, we investigated the sequences of the *MSTN*, *ACVR2A*, and *ACVR2B* genes and determined the interaction between allelic variants and athletic performance and competition level in the Caucasian population. One hundred-two athletes were recruited for the sequencing study, and whole-genome sequencing (WGS) was performed. Second, 330 athletes and 365 controls were included, and real-time PCR was performed.

**Results:**

The sequence analysis revealed two polymorphisms relatively common in the athlete cohort, and the alternate allele showed overrepresentation in athletes: *MSTN* rs11333758 and *ACVR2A* rs3764955. Regarding the polymorphic site *MSTN* rs11333758, there was a significant overrepresentation of the –/– genotype in all high-elite and mixed-sport high-elite athletes. Carriers of the *ACVR2A* rs3764955 CC and GG genotypes were more likely to be elite and high-elite athletes. In addition, carriers of the CC genotype were more likely to be in the mixed-sport subelite group. The gene‒gene interaction analysis revealed that mixed-sport high elite athletes showed significant underrepresentation of the *ACVR2A* rs3764955 GC - *MSTN* rs11333758 AA genotype combination. In the same group, we observed a significant overrepresentation of the *ACVR2A* rs3764955 GC - *MSTN* rs11333758 –/– and the *ACVR2A* rs3764955 CC - *MSTN* rs11333758 –/– genotype combinations.

**Conclusions:**

We showed that the specific genotypes of the *MSTN* rs11333758 and *ACVR2A* rs3764955, either individually or in gene‒gene combination, are significantly associated with athletes’ competition level in the Polish population, especially in the mixed-sports athlete group. Thus, although further research is required, these polymorphisms, alone or in combination with other polymorphisms, are among the numerous candidates that could explain individual variations in muscle phenotypes.

**Supplementary Information:**

The online version contains supplementary material available at 10.1186/s12864-023-09869-2.

## Background

Myostatin, previously known as growth and differentiation Factor 8 (GDF-8), is a member of the transforming growth factor-beta (TGF-β) superfamily. It is highly expressed in skeletal muscle and negatively regulates skeletal muscle growth and development [1]. Previously, investigations have demonstrated that myostatin acts via its receptors, such as activin receptor type IIA (ACVR2A or ActrIIA) and activin receptor type IIB (ACVR2B or ActrIIB), which leads to the recruitment and activation of the suppressor of mothers against decapentaplegic (SMAD) transcription factor family, which regulates the expression of numerous genes. Both receptors are also members of the TGF-ß superfamily [2, 3].

Regarding myostatin, the protein function appears to be conserved across species, since mutations of the myostatin gene (*MSTN*) causing a loss or a reduction in the protein activity have been linked to muscular hypertrophy, which is described in various vertebrate species, including rodents, cattle, dogs, sheep, and cattle [1, 4, 5]. The “double-muscling” phenotypes of animals lacking myostatin and the high degree of sequence conservation during evolution have raised the possibility that myostatin may also control muscle differentiation and growth in humans. Strong evidence that myostatin plays a key role in the regulation of human muscle mass has been provided by Schuelke et al., who described a case of an extraordinarily muscular child with a loss-of-function mutation in the *MSTN* gene [6].

The *MSTN* gene is of interest in sports, where its association with performance, especially in sports that require muscle mass and strength, can be monitored. It could also be used in gene doping in athletes with the aim of increasing muscle mass, strength, and sports performance [7]. Although there is a growing body of evidence explaining the effects of the *MSTN* variation on human muscle mass, athlete studies have been inconclusive. To date, only a few *MSTN* single nucleotide polymorphisms (SNPs), such as K153R (rs1805086, 458 A > G), A55T (rs180565, 163 G > A), E164K (rs35781413, 490 G > A), P198A, I225T, rs11333758 (c.373 + 90delA), and c.373 + 5 G > A (rs397515373), are of particular interest in sports genetics [6, 8–11].

The complications in studying mutations in *MSTN* are the low frequency of some alleles, interethnic differences in allele frequencies, and sex-related differences [9]. Additionally, none of the studies on the strength or endurance abilities of athletes concern the polymorphic sites within genes encoding myostatin receptors (*ACVR2A* and *ACVR2B* genes), which have been described in the context of their effect on growth traits only in chickens [12].

Due to the role of the gene products in regulating skeletal muscle growth and development, the aim of our study was to analyze the sequences of the *MSTN*, *ACVR2A*, and *ACVR2B* genes and determine the interaction between selected allelic variants and athletic performance and competition level in the Caucasian population. Overall, we verify the usefulness of the *MSTN*, *ACVR2A*, and *ACVR2B* genes as genetic markers for sports skills, which may underpin differences in the potential to be an elite athlete. The combination of whole-genome sequencing (WGS), subsequent bioinformatic assessment and validation of selected variants in a larger cohort is a new, comprehensive approach to sport genetics allowing the identification of previously unknown sports-related variants in both coding and noncoding regions of the genome.

## Results

### Genetic variants detected in *MSTN*, *ACVR2A*, and *ACVR2B* with WGS

WGS was performed in a cohort of 102 Polish elite sportsmen and 41 healthy controls from the same population. High-quality SNPs and InDels with at least one non-reference call were identified and yielded 40 variants in *MSTN*, 200 in *ACVR2A*, and 152 in *ACVR2B*. Full results are presented in Supplementary 1 Table A. None of the variants passed the significance threshold after the correction for multiple comparisons when athletes and control groups or athletes subgroups were compared. Additionally, we compared alternate allele frequencies observed in elite athletes with those in the non-Finnish European subset of gnomAD [13] and those in a database of 943 Polish genomes [14]. Here also none of the variants differed significantly in terms of frequency. Nevertheless, based on the obtained results we selected two SNPs for further genotyping: *ACVR2A* rs3764955 and *MSTN* rs11333758 (Table 1). The intronic *ACVR2A* SNP rs3764955 was selected as it was relatively common (minor allele frequency, MAF = 0.27) and the alternate allele showed (non-significant) overrepresentation in the sprint/power subgroups of elite athletes (nominal *p* = 0.027, OR = 2.12). This SNP has a CADD score of 12.9, which is below the commonly-used damaging variant threshold of 16 but is predicted to be within the 5% of most impactful variants, The *MSTN* SNP rs11333758 was selected as, again, it was relatively common in the athletes cohort (MAF = 0.28) and the alternate allele showed (nonsignificant) overrepresentation in elite athletes when compared with non-Finnish European cohort in g-nomAD (nominal *p* = 0.005, OR = 1.57) and a database of Polish genomes (nominal *p* = 0.065, OR = 1.38). Its CADD score is 4.6, meaning that about 35% of all known human SNP variants are more deleterious, which may suggest rather modulatory phenotypic role of this particular polymorphism.

**Table 1:** Characteristics of the selected polymorphic sites

Gene	rsID	*locus*	alleles	CADD	gnomAD MAF	Polish genomes MAF	Predicted consequence
*MSTN*	rs11333758	chr2: 190,062,133	AAA>–AA	12.9	0.2	0.22	Intron variant
*ACVR2A*	rs3764955	chr2: 147,917,228	G > C	4.646	0.31	0.32	Intron variant

CADD - Combined Annotation-Dependent Depletion scores - these scores are phred-scaled values that indicate deleteriousness. CADD 10 means that the variant is within the top 10% of most damaging substitutions in the human genome [15]; MAF - Minor Allele Frequency; gnomAD - genome Aggregation Database

### Genotyping of rs3764955 and rs11333758

Both of the studied SNPs were in Hardy-Weinberg equilibrium (HWE) in the genotyped group (Table 2).

**Table 2:** The overall frequency of genotypes in the study and HWE test results

rs id	*ACVR2A* rs3764955			*MSTN* rs11333758		
**genotype frequency**	GG	CG	CC	AA	A/–	–/–
	0.45 (n = 315)	0.45 (n = 313)	0.1 (n = 67)	0.59 (n = 407)	0.36 (n = 250)	0.05 (n = 38)
**HWE** ***p*** **-value**	0.43			1		

Comparisons of athletes (sprint/power, endurance, and mixed-sport athletes) with controls revealed no significant associations (Supplementary 2 Tables S1 and S2), regardless of assumptions about the genetic model of a trait. Similarly, when athletes were divided into sprint/power, endurance and mixed-sport groups (Supplementary 2 Tables S3 and S4), we found no significant differences in allele frequencies between each discipline and controls in any of the models.

The athletes were also stratified according to their performance: high-elite, elite and subelite (Tables 3 and 4). Here we found a significant underrepresentation of heterozygotes for polymorphism *ACVR2A* rs3764955 in elite athletes (overdominant model corrected *p* = 0.043, Table 3) when compared with the controls. Carriers of the CG genotype were 1.56 times more likely to be in the control group than in the elite athletes group as compared with the CC and GG carriers. Moreover, high-elite athletes showed a similar tendency, with an even higher OR. Heterozygotes for the *ACVR2A* rs3764955 were 1.93 times more likely to be in the control group rather than in the high-elite group as compared with homozygotes. Although, likely due to a smaller n, this association was not significant (overdominant model *p* = 0.067). For the *MSTN* rs11333758 (Table 4) there was a significant overrepresentation of alternate allele homozygotes –/– in the high-elite athletes group (recessive model: OR = 4.39, *p* = 0.004). This SNP also showed significant overrepresentation in high-elite athletes under the codominant model; however, the association was again driven by alternate allele homozygotes (codominant model for deletion genotype carriers: OR = 4.47, *p* = 0.017).

**Table 3:** *ACVR2A* rs3764955 association analysis for athlees stratified by performance and compared with controls

**Model**	**Controls n (%)**	**All high-elite n (%)**	**OR**	**95% CI lower**	**95% CI upper**	***p*** **-value adjusted**
Codominant						
GG	158 (43.3)	29 (56.9)	1			0.20784
CG	179 (49)	17 (33.3)	0.52	0.27	0.98	
CC	28 (7.7)	5 (9.8)	0.97	0.35	2.73	
Dominant						
GG	158 (43.3)	29 (56.9)	1			0.13736
CG - CC	207 (56.7)	22 (43.1)	0.58	0.32	1.05	
Recessive						
GG - CG	337 (92.3)	46 (90.2)	1			1
CC	28 (7.7)	5 (9.8)	1.31	0.48	3.56	
Overdominant						
GG - CC	186 (51)	34 (66.7)	1			0.06678
CG	179 (49)	17 (33.3)	0.52	0.28	0.96	
**Model**	**Controls n (%)**	**All elite n (%)**	**OR**	**95% CI lower**	**95% CI upper**	***p*** **-value adjusted**
Codominant						
GG	158 (43.3)	76 (50.7)	1			0.11374
CG	179 (49)	57 (38)	0.66	0.44	0.99	
CC	28 (7.7)	17 (11.3)	1.26	0.65	2.45	
Dominant						
GG	158 (43.3)	76 (50.7)	1			0.2539
CG - CC	207 (56.7)	74 (49.3)	0.74	0.51	1.09	
Recessive						
GG - CG	337 (92.3)	133 (88.7)	1			0.38214
CC	28 (7.7)	17 (11.3)	1.54	0.82	2.9	
Overdominant						
GG - CC	186 (51)	93 (62)	1			**0.04352**
CG	179 (49)	57 (38)	0.64	0.43	0.94	
**Model**	**Controls n (%)**	**All subelite n (%)**	**OR**	**95% CI lower**	**95% CI upper**	***p*** **-value adjusted**
Codominant						
GG	158 (43.3)	52 (40.3)	1			0.39154
CG	179 (49)	60 (46.5)	1.02	0.66	1.56	
CC	28 (7.7)	17 (13.2)	1.84	0.94	3.64	
Dominant						
GG	158 (43.3)	52 (40.3)	1			1
CG - CC	207 (56.7)	77 (59.7)	1.13	0.75	1.7	
Recessive						
GG - CG	337 (92.3)	112 (86.8)	1			0.14244
CC	28 (7.7)	17 (13.2)	1.83	0.96	3.46	
Overdominant						
GG - CC	186 (51)	69 (53.5)	1			1
CG	179 (49)	60 (46.5)	0.9	0.6	1.35	

**Table 4:** *MSTN* rs11333758 association analysis for athletes stratified by performance and compared with controls

**Model**	**Controls n (%)**	**All high-elite n (%)**	**OR**	**95% CI lower**	**95% CI upper**	***p*** **-value adjusted**
Codominant						
AA	211 (57.8)	25 (49)	1			**0.016968**
A/–	137 (37.5)	17 (33.3)	1.05	0.55	2.01	
–/–	17 (4.7)	9 (17.6)	4.47	1.8	11.08	
Dominant						
AA	211 (57.8)	25 (49)	1			0.474748
A/– - –/–	154 (42.2)	26 (51)	1.42	0.79	2.56	
Recessive						
AA - A/–	348 (95.3)	42 (82.4)	1			**0.004066**
–/–	17 (4.7)	9 (17.6)	4.39	1.84	10.46	
Overdominant						
AA - –/–	228 (62.5)	34 (66.7)	1			1
A/–	137 (37.5)	17 (33.3)	0.83	0.45	1.55	
**Model**	**Controls n (%)**	**All elite n (%)**	**OR**	**95% CI lower**	**95% CI upper**	***p*** **-value adjusted**
Codominant						
AA	211 (57.8)	86 (57.3)	1			1
A/–	137 (37.5)	60 (40)	1.07	0.72	1.59	
–/–	17 (4.7)	4 (2.7)	0.58	0.19	1.77	
Dominant						
AA	211 (57.8)	86 (57.3)	1			1
A/– - –/–	154 (42.2)	64 (42.7)	1.02	0.69	1.5	
Recessive						
AA - A/–	348 (95.3)	146 (97.3)	1			0.56
–/–	17 (4.7)	4 (2.7)	0.56	0.19	1.7	
Overdominant						
AA - –/–	228 (62.5)	90 (60)	1			1
A/–	137 (37.5)	60 (40)	1.11	0.75	1.64	
**Model**	**Controls n (%)**	**All subelite n (%)**	**OR**	**95% CI lower**	**95% CI upper**	***p*** **-value adjusted**
Codominant						
AA	211 (57.8)	85 (65.9)	1			0.25796
A/–	137 (37.5)	36 (27.9)	0.65	0.42	1.02	
–/–	17 (4.7)	8 (6.2)	1.17	0.49	2.81	
Dominant						
AA	211 (57.8)	85 (65.9)	1			0.21014
A/– - –/–	154 (42.2)	44 (34.1)	0.71	0.47	1.08	
Recessive						
AA - A/–	348 (95.3)	121 (93.8)	1			1
–/–	17 (4.7)	8 (6.2)	1.35	0.57	3.22	
Overdominant						
AA - –/–	228 (62.5)	93 (72.1)	1			0.09222
A/–	137 (37.5)	36 (27.9)	0.64	0.42	1	

Finally, the athletes were divided both by their discipline type and athletic performance (Tables 5 and 6). For clarity, only results with at least one significant association are presented and full results for both SNPs are available in Supplementary 1 Table B. For *ACVR2A* rs3764955, heterozygotes were 2.12 times less likely to be sprint/power elite athletes than controls (overdominant model *p* = 0.048, Table 5), while carriers for the alternate homozygous genotype (CC) were 2.65 times more likely to be in the mixed-sport subelite group as compared to the control group. In the case of *MSTN* rs11333758 (Table 6) mixed-sport high-elite athletes showed significant overrepresentation of the alternate genotype (codominant model *p* = 0.002). In the codominant model, heterozygotes (A/–) had genotypic OR of 3,1 and homozygotes (–/–) of 15.5. This association was also significant for both the recessive and dominant models (dominant model *p* = 0.01, recessive model *p* = 0.0027).

**Table 5:** The results of association analysis for the *ACVR2A* rs3764955 with athletes stratified by both discipline and performance. Full results are available in Supplementary 1 Table B

**Model**	**Controls n (%)**	**Sprint/power elite n (%)**	**OR**	**95% CI lower**	**95% CI upper**	***p*** **-value adjusted**
Codominant						
GG	158 (43.3)	22 (52.4)	1			0.07862
CG	179 (49)	13 (31)	0.52	0.25	1.07	
CC	28 (7.7)	7 (16.7)	1.8	0.7	4.6	
Dominant						
GG	158 (43.3)	22 (52.4)	1			0.52572
CG - CC	207 (56.7)	20 (47.6)	0.69	0.37	1.32	
Recessive						
GG - CG	337 (92.3)	35 (83.3)	1			0.1464
CC	28 (7.7)	7 (16.7)	2.41	0.98	5.91	
Overdominant						
GG - CC	186 (51)	29 (69)	1			**0.04824**
CG	179 (49)	13 (31)	0.47	0.23	0.92	
**Model**	**Controls n (%)**	**Mixed-sport sub-elite n (%)**	**OR**	**95% CI lower**	**95% CI upper**	***p*** **-value adjusted**
Codominant						
GG	158 (43.3)	24 (39.3)	1			0.11748
CG	179 (49)	26 (42.6)	0.96	0.53	1.73	
CC	28 (7.7)	11 (18)	2.59	1.14	5.87	
Dominant						
GG	158 (43.3)	24 (39.3)	1			1.12636
CG - CC	207 (56.7)	37 (60.7)	1.18	0.68	2.05	
Recessive						
GG - CG	337 (92.3)	50 (82)	1			**0.03496**
CC	28 (7.7)	11 (18)	2.65	1.24	5.65	
Overdominant						
GG - CC	186 (51)	35 (57.4)	1			0.70418
CG	179 (49)	26 (42.6)	0.77	0.45	1.33	

**Table 6:** The results of association analysis for *MSTN* rs11333758 with athletes stratified by both discipline and performance. Full results are available in Supplementary 1 Table B

Model	Controls n (%)	Mixed-sport high elite n (%)	OR	95% CI lower	95% CI upper	*p*-value adjusted
Codominant						
AA	211 (57.8)	4 (23.5)	1			**0.0020584**
A/–	137 (37.5)	8 (47.1)	3.08	0.91	10.43	
–/–	17 (4.7)	5 (29.4)	15.51	3.81	63.2	
Dominant						
AA	211 (57.8)	4 (23.5)	1			**0.009847**
A/– - –/–	154 (42.2)	13 (76.5)	4,45	1,42	13,92	
Recessive						
AA - A/–	348 (95.3)	12 (70.6)	1			**0.0027478**
–/–	17 (4.7)	5 (29.4)	8.53	2.7	26.97	
Overdominant						
AA - –/–	228 (62.5)	9 (52.9)	1			0.8686588
A/–	137 (37.5)	8 (47.1)	1.48	0.56	3.92	

### *ACVR2A* rs3764955 and *MSTN* rs11333758 interaction analysis

Although both selected SNPs are located on the same chromosome 2, their distance of over 40 million bp is past the distance (~ 500 000 bp) at which linkage disequilibrium (LD) in the human genome decays completely [16]. Therefore, they cannot be considered as a haplotype. Here we tested for interaction between *MSTN* rs3764955 and *ACVR2A* rs11333758 using the model-free W-test for epistasis. *p*-values for each comparison are reported in Supplementary 1 Table C. The group of mixed-sport high-elite athletes showed a significant interaction between the tested SNPs (*p* = 0.0057). Table 7 shows the frequencies of each genotype combination in these two groups. The largest difference was observed in the between carriers of the *ACVR2A* rs3764955 GC and *MSTN* rs11333758 AA combination. In controls, this combination had a frequency of 0.28 (n = 104) while none of the mixed-sport high-elite athletes was a carrier of this genotype set (Fisher exact *p* = 0.005). On the other hand, in the mixed-sport high-elite group, we observed a significant overrepresentation of carriers of the *ACVR2A* rs3764955 GC with *MSTN* rs11333758 –/– combination (Fisher exact *p* = 0.0097) and the *ACVR2A* rs3764955 CC with *MSTN* rs11333758 –/– (Fisher exact *p* = 0.0445).

**Table 7:** Frequencies of different genotype combinations in mixed high-elite athletes and controls. In this comparison, a significant epistasis between the genotypes was found (Supplementary 1 Table C). Fisher exact test was run for each combination

**rs3764955 genotype**	GG	GC	CC	GG	GC	CC	GG	GC	CC
**rs11333758 genotype**	AA	AA	AA	A/–	A/–	A/–	–/–	–/–	–/–
**group**	**Mixed-sport high-elite**								
**n**	0.23 (n = 4)	0	0	0.35 (n = 6)	0.12 (n = 2)	0	0.06 (n = 1)	0.18 (n = 3)	0.06 (n = 1)
**group**	**controls**								
**n**	0.24 (n = 88)	0.28 (n = 104)	0.05 (n = 19)	0.17 (n = 61)	0.18 (n = 67)	0.025 (n = 9)	0.025 (n = 9)	0.022 (n = 8)	0
**Fisher exact** ***p*** **-value**	1	**0.005**	1	0.093	0.748	1	0.369	**0.0097**	**0.0445**

## Discussion

Skeletal muscle size, which is crucial to athletes’ strength, is one of the most heritable quantitative traits in humans, with genetic variation contributing 92–94% of the total variance in muscle circumference [17]. While myostatin is an important factor influencing muscle mass, which is well confirmed in nonhuman species, it has not been clearly confirmed whether *MSTN* expression influences interindividual differences in skeletal muscle mass, affects posttraining changes in body composition, or plays a role in the age-related loss of skeletal muscle mass and function in humans [11]. Although the inconclusive results are usually explained by ethnic differences and the low frequency of some alleles [18], it is possible that the role of receptors that affect the biological activity of myostatin is crucial. However, no study has analyzed the association of the polymorphic sites not only within the *MSTN* gene but also within the genes encoding its receptors with interindividual differences in sports abilities. Therefore, we conducted a comprehensive study sequencing the *MSTN*, *ACVR2A*, and *ACVR2B* genes from 102 elite athletes and 41 controls. The sequence analysis revealed two nonsynonymous polymorphisms (rs11333758 in *MSTN* and rs3764955 in *ACVR2*) relatively common in the athlete cohort, and the alternate allele showed overrepresentation in elite athletes. The first polymorphism was the deletion of one of three adenines (AAA→AA) at position 88–90 bp in the first intron of *MSTN* (c.373 + 90delA, rs11333758). The second was rs3764955, located at the end of the fifth intron of *ACVR2*, involving a G to C transversion. Selected SNPs are relatively common in the European population and are not expected to be pathogenic.

The comparisons of all athletes with the controls and the comparisons of athletes divided into sprint/power, endurance, and mixed-sport groups with the controls revealed no significant associations in any of the models. However, we found significant differences when the athletes were stratified according to their competition level: high-elite, elite, and subelite. Regarding the polymorphic site rs11333758 located in *MSTN*, there was a significant overrepresentation of rare homozygotes (–/–) in the high-elite athletes group when compared with the controls. In addition, when the athletes were divided by their discipline type and athletic performance, mixed-sport high-elite athletes showed significant overrepresentation of the –/– genotype. This finding suggests that this genotype may be favorable for achieving success in sports utilizing mixed anaerobic/aerobic energy production. Thus, for the first time, this experiment revealed that harboring this indel *MSTN* variant is significantly associated with athletes’ competition level in the Polish population, especially in the mixed-sport group. To our knowledge, only three studies have been published to date on the functional relevance of the rs11333758 polymorphism in the *MSTN* gene [19, 20]. First, in a study including 110 elite athletes with a high amount of endurance training and 27 male controls, Karlowatz et al. (2011) analyzed the association of the left ventricular mass (LVM) of an athlete’s heart with polymorphisms in the insulin-like growth Factor 1 (IGF1) signaling pathway in combination with MSTN. An analysis of the *MSTN* sequence revealed only one significant polymorphism, rs11333758, in the *MSTN* gene. An increased *MSTN* effect for the deletion allele was observed. Thus, the carriers of the A/– and –/– genotypes may show an attenuated training-induced growth response of the heart, resulting in a lesser LVM increase in comparison with carriers homozygous for the wild type allele (A/A) [20]. Second, Gineviciene et al. (2021) analyzed the whole coding sequence of the *MSTN* gene in a group of 103 Lithuanian elite athletes and 127 controls. They confirmed an association between the rs11333758 polymorphism and elite athlete status, suggesting that this SNP affects the development of physical performance phenotypes. Specifically, the associations of the deletion allele and genotype with success in endurance sports in female athletes and in sprint/power-oriented male athletes were demonstrated [19]. Third, *MSTN* sequence analysis performed by Al Majidi et al. (2022) revealed that the homozygous deletion genotype (–/–) was significantly higher in Iraqi endurance athletes and power athletes than in the controls [21]. It was suggested that despite intronic and not altering the amino acid composition of myostatin, this variant may affect the expression of the *MSTN* gene and myostatin function [19, 20]. Our study and previous data confirmed a potential role of this polymorphism in determining the success of elite athletes; however, more experimental data are required.

It should be emphasized that the rs3764955 polymorphism in *ACVR2A* has not been previously described in athletes; thus, our results cannot be discussed in direct comparison with other studies. The performed analysis revealed a significant underrepresentation of rs3764955 GC heterozygotes in elite athletes when compared with that in the controls. Carriers of the CC and GG genotypes were more likely than heterozygotes to be elite athletes. Moreover, high-elite athletes showed a similar tendency. In addition, when the athletes were divided both by their discipline type and athletic performance, GC heterozygotes were less likely than the controls to be sprint/power elite athletes, while carriers for the CC genotype were more likely than the controls to be in the mixed-sport subelite group. These results suggest that the GC genotype may be unfavorable regarding achieving success in sports. In humans, the rs3764955 polymorphism was only described in the context of the risk of hypertensive disorders of pregnancy in the northern Chinese population [22] and preeclampsia in the Norwegian population [23].

Earlier investigations demonstrated that myostatin negatively regulates skeletal muscle development by activating its receptors [3]. In an animal study, Bhattacharya et al. demonstrated that a single knockdown of *MSTN* and its receptors could enhance growth traits more so than combinations of *MSTN* and its receptors [12]. Thus, only a simultaneous analysis of polymorphic sites located in *MSTN* and genes encoding myostatin receptors can provide additional unique information about the relationships between the gene variants and observed phenotypic traits and insight into the dependency among genetic markers [24]. To the best of our knowledge, this is the first study to analyze the association of the *MSTN*-*ACVR2A* interaction with athletic performance and competition level. The complex analysis revealed that mixed-sport high-elite athletes showed significant underrepresentation of the rs3764955 GC - rs11333758 AA genotype combination (none of the mixed high-elite athletes was a carrier of this genotype set). We observed a significant overrepresentation of the rs3764955 GC - rs11333758 –/– and rs3764955 CC - rs11333758 –/– genotype combination in the same group. Thus, it can be concluded that the rs3764955 GC - rs11333758 AA genotype combination might be unfavorable for achieving success in mixed-sports. In addition, the rs3764955 GC - rs11333758 –/– and rs3764955 CC - rs11333758 –/– genotype combinations may be considered beneficial, and carriers of these combinations of genotypes might achieve sports success utilizing mixed anaerobic/aerobic energy production. The results of gene‒gene interaction analyses confirmed the results of individual SNP analysis.

Although myostatin is usually described as a key factor affecting the growth and differentiation of muscle cells, which is advantageous in the improvement of strength and power [10, 11], animal studies have revealed that the effects of myostatin on the mechanical properties of muscles depend on species, muscle type, developmental stage, and the extra and intracellular factors determining the fiber type [5, 25]. These factors may partly explain the inconsistent findings regarding myostatin [8]. Interestingly, it has been shown that myostatin is expressed at higher levels in slow-twitch muscle fibers and may therefore have a more significant functional impact in this muscle group [26], which explains the described association between the rs11333758 *MSTN* polymorphisms and endurance predispositions in female athletes [19]. An association between this SNP and success has also been demonstrated in sprint/power-oriented male athletes, indicating a sex difference in the effect of the rs11333758 SNP on athletes’ physical performance [19]. A systematic large-scale rare-variant association analysis of 4,529 phenotypes revealed that missense variants in *MSTN* are associated with body composition and creatinine levels [27]. We did not compare genotype frequencies with respect to sex; thus, we cannot confirm this relationship. We found an association between the specific *MSTN* rs11333758 and *ACVR2A* rs3764955 genotypes with the mixed-sport group, designated strength-endurance athletes, comprising athletes whose sports utilize mixed anaerobic/aerobic energy production. Unfortunately, the athletes in the other studies were stratified into only two groups (endurance-oriented and sprint/power-oriented athletes), complicating the comparison of results [19, 21]. Although the important role of myostatin in skeletal muscle development has been confirmed, additional replication studies are needed to establish its role in sports performance. In addition, epigenetic mechanisms that determine whether a gene is silenced or activated and when, and in what tissue it will be expressed, should be considered [28].

## Conclusions

The sequence analysis performed in the present study revealed two polymorphisms (rs11333758 in *MSTN* and rs3764955 in *ACVR2A*) likely associated with sports abilities. We then confirmed that the specific genotypes of the selected SNPs, either individually or in gene‒gene combination, are significantly associated with athletes’ competition level in the Polish population, especially in the mixed-sports athlete group (strength-endurance athletes). Thus, although further research is required, these polymorphisms, alone or in combination with other polymorphisms, are among the numerous candidates that could explain individual variations in muscle phenotypes.

## Materials and methods

### Participants

In the first part of the experiment involving WGS, the study group consisted of 101 male and 1 female elite sprint/power (n = 53) and endurance (n = 49) athletes (age: 23.5 ± 5.9 years) of the highest nationally-competitive standards (classification was based on scoring tables, e.g., International Amateur Athletic Federation (IAAF), International Federation of Swimming (FINA), or receiving a medal in the national championships, or participation in international competition at the European or World Championships). Athletes with personal best results ranking them in the top 100 in a particular sports discipline in the world or in Europe were included in the study group. As the aim of this part of the study was to determine genetic variants associated with overall physical performance, all athletes were considered as a single group.

The control group consisted of 41 healthy individuals (23 females and 18 males) with no pairs with kinship above 0.125. Kinship was assessed with Hail (pc_relate method [29]; kinship metric scale 0–0.5; age: 22.4 ± 6.3 years). The inclusion criteria for volunteers were no medical history of any cardiorespiratory diseases, and not participating in professional sport training.

The second part of our study aimed to assess the association of selected gene variants with sport-related phenotypes, and 330 Polish athletes (age: 27.8 ± 7.1 years; 82 females and 248 males) who competed in national and international events were involved. The athletes were stratified into three groups according to values of relative anaerobic/aerobic energy system contribution, time of competitive exercise performance, and intensity of exertion in each sport:

- endurance athletes (n = 101): rowers (n = 36), 1500–3000 m swimmers (n = 13), 15–50 km cross-country skiers (n = 4), canoeing (n = 9), road cyclists (n = 10), 1500 m runners (n = 9), 3–10 km runners (n = 8), triathletes (n = 6), marathon runners (n = 6);
- mixed-sport athletes (n = 138): fencers (n = 10), boxers (n = 16), judokas (n = 11), wrestlers (n = 37), karate fighters (n = 1), volleyball players (n = 13), handball players (n = 21), ice hockey players (n = 25), gymnasts (n = 1), pentathletes (n = 3);
- sprint/power athletes (n = 91): jumpers (n = 8), 100–400 m runners (n = 29), weightlifters (n = 17), powerlifters (n = 19), archers (n = 4), throwers (n = 5), 50–100 m swimmers (n = 9).

The athletes in these groups were divided into subgroups according to their competition level: high-elite (n = 51; gold medalists in the World and European Championships, World Cups, or Olympic Games), elite (n = 150; silver or bronze medalists in the World and European Championships, World Cups, or Olympic Games), and subelite (n = 129; participants in international competitions).

Non-training unrelated students (n = 365) from the Gdansk University of Physical Education and Sport (age 22 ± 3.4 years; 153 females and 212 males) were included in the control group. Athletes in the first and second part of the study, as well as controls, were of Polish origin.

We performed sample size calculations to assess the power of the genotyping study using a tool available at RE: https://clincalc.com/stats/samplesize.aspx, and assumed the following parameters: power 90%, effect size 25%, and alpha 0.05. The results indicated a minimum group size of 329 participants per group.

### WGS data processing and analysis

DNA was isolated from peripheral blood leukocytes using the standard salting-out procedure and from saliva using the Oragene DNA self-collection kit and Prep IT L2P Purification Kit (DNA Genotek Inc., Stittsville, ON, Canada) following the manufacturer’s instructions.

All WGS data analyses were performed with Hail (v. 0.2.85) [29]. WGS and quality control (QC) was performed as described previously [30]. For this study, after QC whole-genome .vcf files were filtered to contain variants only in *MSTN* (40 variants), *ACVR2A* (200 variants), and *ACVR2B* (152 variants). Fisher exact tests were used to compare allele frequencies of sequenced variants between athletes and controls, sportsmen subgroups (endurance vs. sprint/power) and the sportsmen group with two external control groups: non-Finnish European population from gnomAD database with data from 33 988 samples [13] and a database of 1000 Polish genomes with data from 943 samples [31]. Bonferroni correction was applied to the obtained *p*-values and corrected *p*-values are reported in this manuscript unless clearly stated otherwise. The code used to export, annotate and test variants in *MSTN*, *ACVR2A*, and *ACVR2B* is available in the projects’ GitHub repository [32].

### SNPs genotyping

DNA was extracted from buccal cells with a GenElute Mammalian Genomic DNA Miniprep Kit (Sigma, Steinheim, Germany) in accordance with the manufacturer’s protocol. All samples were genotyped using an allelic discrimination assay on a C1000 Touch Thermal Cycler (Bio–Rad, Feldkirchen, Germany) instrument with TaqMan® probes. To differentiate the *MSTN* rs11333758 and *ACVR2A* rs3764955 alleles, TaqMan® Pre-Designed SNP Genotyping Assays (Applied Biosystems, Waltham, MA, U.S.A.; assay ID: C_175825166_10 and C___3144655_10, respectively) containing fluorescently labeled (VIC and FAM) probes and primers were used.

### Statistical analyses of genotyping results

All analyses were performed in R studio 4.2.0 (R Core Team 2020, R: A language and environment for statistical computing, R Foundation for Statistical Computing, Vienna, Austria, [33]). SNPassoc R package was used for single-SNP and Hardy–Weinberg equilibrium tests. Association tests were performed for the following genetic models: codominant, dominant, recessive, and overdominant. For each comparison, odds ratio (OR), *p*-value, and 95% confidence interval (CI) are reported. SNP epistasis was tested with W-test in the wtest R package [34]. All reported *p*-values were corrected for the number of variants genotyped using the Bonferroni correction. *p*-values < 0.05 were considered significant.

### Electronic supplementary material

Below is the link to the electronic supplementary material.

Supplementary Material 1

Supplementary Material 2

## Data Availability

The datasets generated and/or analyzed during the current study are available in the OSF repository, DOI 10.17605/OSF.IO/6UQ9E. The datasets generated and/or analyzed during the current study as well as Supplementary 1 Tabels A, B, C and Supplementary 2 Tables S1, S2, S3, S4 are available in the OSF repository, DOI 10.17605/OSF.IO/6UQ9E.

## Abbreviations

ACVR2A or ActrIIA: Activin receptor type IIA
ACVR2B or ActrIIB: Activin receptor type IIB
AF: Allele frequency
CADD: Combined annotation-dependent depletion
CI: Confidence interval
GDF-8: Growth and differentiation Factor 8
gnomAD: Genome Aggregation Database
HWE: Hardy-Weinberg equilibrium
IGF1: Insulin-like growth Factor 1
LD: Linkage disequilibrium
LVM: Left ventricular mass
MAF: Minor allele frequency
MSTN: Myostatin
OR: Odds ratio
SNPs: Single nucleotide polymorphisms
SMAD: Suppressor of mothers against decapentaplegic
TGF-β: Transforming growth factor-beta
WGS: Whole-genome sequencing
QC: Quality control
