# Correction: Genetic variants in myostatin and its receptors promote elite athlete status

**DOI:** 10.1186/s12864-023-09947-5

**Published:** 2024-01-03

**Authors:** Agata Leońska-Duniec, Małgorzata Borczyk, Michał Korostyński, Myosotis Massidda, Ewelina Maculewicz, Paweł Cięszczyk

**Affiliations:** 1 Faculty of Physical Education, Gdansk University of Physical Education and Sport, 80-336 Gdansk, Poland; 2 Department of Medical Sciences and Public Health, University of Cagliari, 09124 Cagliari, Italy; 3 Laboratory of Pharmacogenomics, Department of Molecular Neuropharmacology, Maj Institute of Pharmacology, Polish Academy of Sciences, 31-343 Cracow, Poland; 4 Faculty of Physical Education, Jozef Pilsudski University of Physical Education in Warsaw, 00-809 Warsaw, Poland

**Correction: BMC Genomics 24, 761 (2023)**

10.1186/s12864-023-09869-2

Following publication of the original article [1], it was reported that Ewelina Maculewicz was erroneously assigned affiliation 1. The original article has been updated with the accurate affiliations listed.
