# Supplementary material for: Genetic variants in myostatin and its receptors promote elite athlete status
Source: BMC Genomics. 2023 Dec 11;24:761. doi: 10.1186/s12864-023-09869-2 (PMC10712039; doi:10.1186/s12864-023-09869-2)
Supplement: Supplementary file 2 — Supplementary Material 2 [file 12864_2023_9869_MOESM2_ESM.docx]

Table S1. Genetic association analysis of *ACVR2A* rs3764955 variant for all sports and controls.

| **Model** | **Controls**  **n (%)** | **All sports**  **n (%)** | **OR** | **95% CI lower** | **95% CI upper** | **p-value adjusted** |
| --- | --- | --- | --- | --- | --- | --- |
| Codominant |  |  |  |  |  |  |
| GG | 158 (43.3) | 157 (47.6) | 1 |  |  | 0.07578 |
| CG | 179 (49) | 134 (40.6) | 0.75 | 0.55 | 1.03 |  |
| CC | 28 (7.7) | 39 (11.8) | 1.4 | 0.82 | 2.39 |  |
| Dominant |  |  |  |  |  |  |
| GG | 158 (43.3) | 157 (47.6) | 1 |  |  | 0.51356 |
| CG - CC | 207 (56.7) | 173 (52.4) | 0.84 | 0.62 | 1.13 |  |
| Recessive |  |  |  |  |  |  |
| GG - CG | 337 (92.3) | 291 (88.2) | 1 |  |  | 0.12856 |
| CC | 28 (7.7) | 39 (11.8) | 1.61 | 0.97 | 2.69 |  |
| Overdominant |  |  |  |  |  |  |
| GG - CC | 186 (51) | 196 (59.4) | 1 |  |  | 0.05098 |
| CG | 179 (49) | 134 (40.6) | 0.71 | 0.53 | 0.96 |  |

Table S2. Genetic association analysis of *MSTN* rs11333758 variant for all sports and controls.

| **Model** | **Controls**  **n (%)** | **All sports**  **n (%)** | **OR** | **95% CI lower** | **95% CI upper** | **p-value**  **adjusted** |
| --- | --- | --- | --- | --- | --- | --- |
| Codominant |  |  |  |  |  |  |
| AA | 211 (57.8) | 196 (59.4) | 1 |  |  | 0.9358 |
| A/- | 137 (37.5) | 113 (34.2) | 0.89 | 0.65 | 1.22 |  |
| -/- | 17 (4.7) | 21 (6.4) | 1.33 | 0.68 | 2.59 |  |
| Dominant |  |  |  |  |  |  |
| AA | 211 (57.8) | 196 (59.4) | 1 |  |  | 1 |
| A/– - –/– | 154 (42.2) | 134 (40.6) | 0.94 | 0.69 | 1.27 |  |
| Recessive |  |  |  |  |  |  |
| AA - A/– | 348 (95.3) | 309 (93.6) | 1 |  |  | 0.647 |
| –/– | 17 (4.7) | 21 (6.4) | 1.39 | 0.72 | 2.69 |  |
| Overdominant |  |  |  |  |  |  |
| AA - –/– | 228 (62.5) | 217 (65.8) | 1 |  |  | 0.7326 |
| A/– | 137 (37.5) | 113 (34.2) | 0.87 | 0.64 | 1.18 |  |

Table S3. *ACVR2A* rs3764955 association analysis for athletes divided by discipline compared with controls.

| **Model** | **Controls**  **n (%)** | **Endurance**  **n (%)** | **OR** | **95% CI lower** | **95% CI upper** | **p-value**  **adjusted** |
| --- | --- | --- | --- | --- | --- | --- |
| Codominant |  |  |  |  |  |  |
| GG | 158 (43.3) | 48 (47.5) | 1 |  |  | 1 |
| CG | 179 (49) | 45 (44.6) | 0.83 | 0.52 | 1.31 |  |
| CC | 28 (7.7) | 8 (7.9) | 0.94 | 0.4 | 2.2 |  |
| Dominant |  |  |  |  |  |  |
| GG | 158 (43.3) | 48 (47.5) | 1 |  |  | 0.8974 |
| CG - CC | 207 (56.7) | 53 (52.5) | 0.84 | 0.54 | 1.31 |  |
| Recessive |  |  |  |  |  |  |
| GG - CG | 337 (92.3) | 93 (92.1) | 1 |  |  | 1 |
| CC | 28 (7.7) | 8 (7.9) | 1.04 | 0.46 | 2.35 |  |
| Overdominant |  |  |  |  |  |  |
| GG - CC | 186 (51) | 56 (55.4) | 1 |  |  | 0.848 |
| CG | 179 (49) | 45 (44.6) | 0.83 | 0.54 | 1.3 |  |
| **Model** | **Controls**  **n (%)** | **Sprint/power**  **n (%)** | **OR** | **95% CI lower** | **95% CI upper** | **p-value**  **adjusted** |
| Codominant |  |  |  |  |  |  |
| GG | 158 (43.3) | 47 (51.6) | 1 |  |  | 0.13906 |
| CG | 179 (49) | 33 (36.3) | 0.62 | 0.38 | 1.02 |  |
| CC | 28 (7.7) | 11 (12.1) | 1.32 | 0.61 | 2.85 |  |
| Dominant |  |  |  |  |  |  |
| GG | 158 (43.3) | 47 (51.6) | 1 |  |  | 0.30468 |
| CG - CC | 207 (56.7) | 44 (48.4) | 0.71 | 0.45 | 1.13 |  |
| Recessive |  |  |  |  |  |  |
| GG - CG | 337 (92.3) | 80 (87.9) | 1 |  |  | 0.3905 |
| CC | 28 (7.7) | 11 (12.1) | 1.65 | 0.79 | 3.46 |  |
| Overdominant |  |  |  |  |  |  |
| GG - CC | 186 (51) | 58 (63.7) | 1 |  |  | 0.05548 |
| CG | 179 (49) | 33 (36.3) | 0.59 | 0.37 | 0.95 |  |
| **Model** | **Controls**  **n (%)** | **Mixed-sport**  **n (%)** | **OR** | **95% CI lower** | **95% CI upper** | **p-value**  **adjusted** |
| Codominant |  |  |  |  |  |  |
| GG | 158 (43.3) | 62 (44.9) | 1 |  |  | 0.09326 |
| CG | 179 (49) | 56 (40.6) | 0.8 | 0.52 | 1.21 |  |
| CC | 28 (7.7) | 20 (14.5) | 1.82 | 0.96 | 3.47 |  |
| Dominant |  |  |  |  |  |  |
| GG | 158 (43.3) | 62 (44.9) | 1 |  |  | 1 |
| CG - CC | 207 (56.7) | 76 (55.1) | 0.94 | 0.63 | 1.39 |  |
| Recessive |  |  |  |  |  |  |
| GG - CG | 337 (92.3) | 118 (85.5) | 1 |  |  | 0.05038 |
| CC | 28 (7.7) | 20 (14.5) | 2.04 | 1.11 | 3.76 |  |
| Overdominant |  |  |  |  |  |  |
| GG - CC | 186 (51) | 82 (59.4) | 1 |  |  | 0.17764 |
| CG | 179 (49) | 56 (40.6) | 0.71 | 0.48 | 1.06 |  |

Table S4. *MSTN* rs11333758 association analysis for athletes divided by discipline compared with controls.

| **Model** | **Controls**  **n (%)** | **Endurance**  **n (%)** | **OR** | **95% CI lower** | **95% CI upper** | **p-value**  **adjusted** |
| --- | --- | --- | --- | --- | --- | --- |
| Codominant |  |  |  |  |  |  |
| AA | 211 (57.8) | 63 (62.4) | 1 |  |  | 1 |
| A/– | 137 (37.5) | 34 (33.7) | 0.83 | 0.52 | 1.33 |  |
| –/– | 17 (4.7) | 4 (4) | 0.79 | 0.26 | 2.43 |  |
| Dominant |  |  |  |  |  |  |
| AA | 211 (57.8) | 63 (62.4) | 1 |  |  | 0.8148 |
| A/– - –/– | 154 (42.2) | 38 (37.6) | 0.83 | 0.53 | 1.3 |  |
| Recessive |  |  |  |  |  |  |
| AA - A/– | 348 (95.3) | 97 (96) | 1 |  |  | 1 |
| –/– | 17 (4.7) | 4 (4) | 0.84 | 0.28 | 2.57 |  |
| Overdominant |  |  |  |  |  |  |
| AA - –/– | 228 (62.5) | 67 (66.3) | 1 |  |  | 0.946 |
| A/– | 137 (37.5) | 34 (33.7) | 0.84 | 0.53 | 1.34 |  |
| **Model** | **Controls**  **n (%)** | **Sprint/power**  **n (%)** | **OR** | **95% CI lower** | **95% CI upper** | **p-value**  **adjusted** |
| Codominant |  |  |  |  |  |  |
| AA | 211 (57.8) | 53 (58.2) | 1 |  |  | 1 |
| A/– | 137 (37.5) | 33 (36.3) | 0.96 | 0.59 | 1.56 |  |
| –/– | 17 (4.7) | 5 (5.5) | 1.17 | 0.41 | 3.32 |  |
| Dominant |  |  |  |  |  |  |
| AA | 211 (57.8) | 53 (58.2) | 1 |  |  | 1 |
| A/– - –/– | 154 (42.2) | 38 (41.8) | 0.98 | 0.62 | 1.56 |  |
| Recessive |  |  |  |  |  |  |
| AA - A/– | 348 (95.3) | 86 (94.5) | 1 |  |  | 1 |
| –/– | 17 (4.7) | 5 (5.5) | 1.19 | 0.43 | 3.32 |  |
| Overdominant |  |  |  |  |  |  |
| AA - –/– | 228 (62.5) | 58 (63.7) | 1 |  |  | 1 |
| A/– | 137 (37.5) | 33 (36.3) | 0.95 | 0.59 | 1.53 |  |
| **Model** | **Controls**  **n (%)** | **Mixed-sport**  **n (%)** | **OR** | **95% CI lower** | **95% CI upper** | **p-value**  **adjusted** |
| Codominant |  |  |  |  |  |  |
| AA | 211 (57.8) | 80 (58) | 1 |  |  | 042394 |
| A/– | 137 (37.5) | 46 (33.3) | 0.89 | 0.58 | 1.35 |  |
| –/– | 17 (4.7) | 12 (8.7) | 1.86 | 0.85 | 4.07 |  |
| Dominant |  |  |  |  |  |  |
| AA | 211 (57.8) | 80 (58) | 1 |  |  | 1 |
| A/– - –/– | 154 (42.2) | 58 (42) | 0.99 | 0.67 | 1.48 |  |
| Recessive |  |  |  |  |  |  |
| AA - A/– | 348 (95.3) | 126 (91.3) | 1 |  |  | 0.19066 |
| –/– | 17 (4.7) | 12 (8.7) | 1.95 | 0.91 | 4.2 |  |
| Overdominant |  |  |  |  |  |  |
| AA - –/– | 228 (62.5) | 92 (66.7) | 1 |  |  | 0.7606 |
| A/– | 137 (37.5) | 46 (33.3) | 0.83 | 0.55 | 1.26 |  |
